# Reorganization of effective network structure with dynamic synapses in cortical circuit and its possible functions

**DOI:** 10.1186/1471-2202-14-S1-P4

**Published:** 2013-07-08

**Authors:** Yuichi Katori, Kazuhiro Sakamoto, Hajime Mushiake, Kazuyuki Aihara

**Affiliations:** 1 FIRST, Aihara Innovative Mathematical Modelling Project, JST, Institute of Industrial Science, The University of Tokyo, 4-6-1-Cw601 Komaba, Meguro-ku, Tokyo 153-8505, Japan; 2 Institute of Industrial Science, The University of Tokyo, Japan; 3 Research Institute of Electrical Communication, Tohoku University, 2-1-1 Katahira, Aoba-ku, Sendai 980-8577, Japan; 4 Department of Physiology, Tohoku University School of Medicine, 2-1 Seiryo-machi Aoba-Ku, Sendai 980-8575 Japan

## 

Synaptic transmission efficacy transiently changes in a short period of time with generation of presynaptic spikes. Depending on changes in releasable neurotransmitters and calcium concentration in presynaptic terminals, the transmission efficacy of the dynamic synapses decreases (short-term depression) or increases (short-term facilitation) [1]. Dynamical properties of the neural network with dynamic synapses have been intensively investigated [1-3]. In the associative memory network with dynamic synapses, the network exhibits not only memory retrieved state but also state transitions among stored memory patterns [2]. Further, we propose that the changes in the synaptic transmission efficacy cause the reorganization of effective network structure and, thereby functions of the network changes dynamically according to a required task [3].

We investigate the properties of the network dynamics with the leaky integrate-and-fire based spiking neural network and the corresponding mean field model. Changes in the synaptic transmission efficacy can be modeled with variables that represent the releasable neurotransmitter and the utilization parameter reflecting the calcium concentration. In the case of the facilitation synapses, the transmission efficacy increases with the successive generation of presynaptic spikes as shown in Figure 1A. Activation of a sub-network facilitates the synaptic connections between the neurons in the sub-network (Figure 1B). We drive the mean field model that captures its population dynamics. Changes in the synaptic efficacy are relatively slow, and the slow variables can be regarded as bifurcation parameters that have influences on the fast variables of the neural activity. On the basis of this concept, we analyze bifurcation structure of the mean field model.

**Figure 1:**
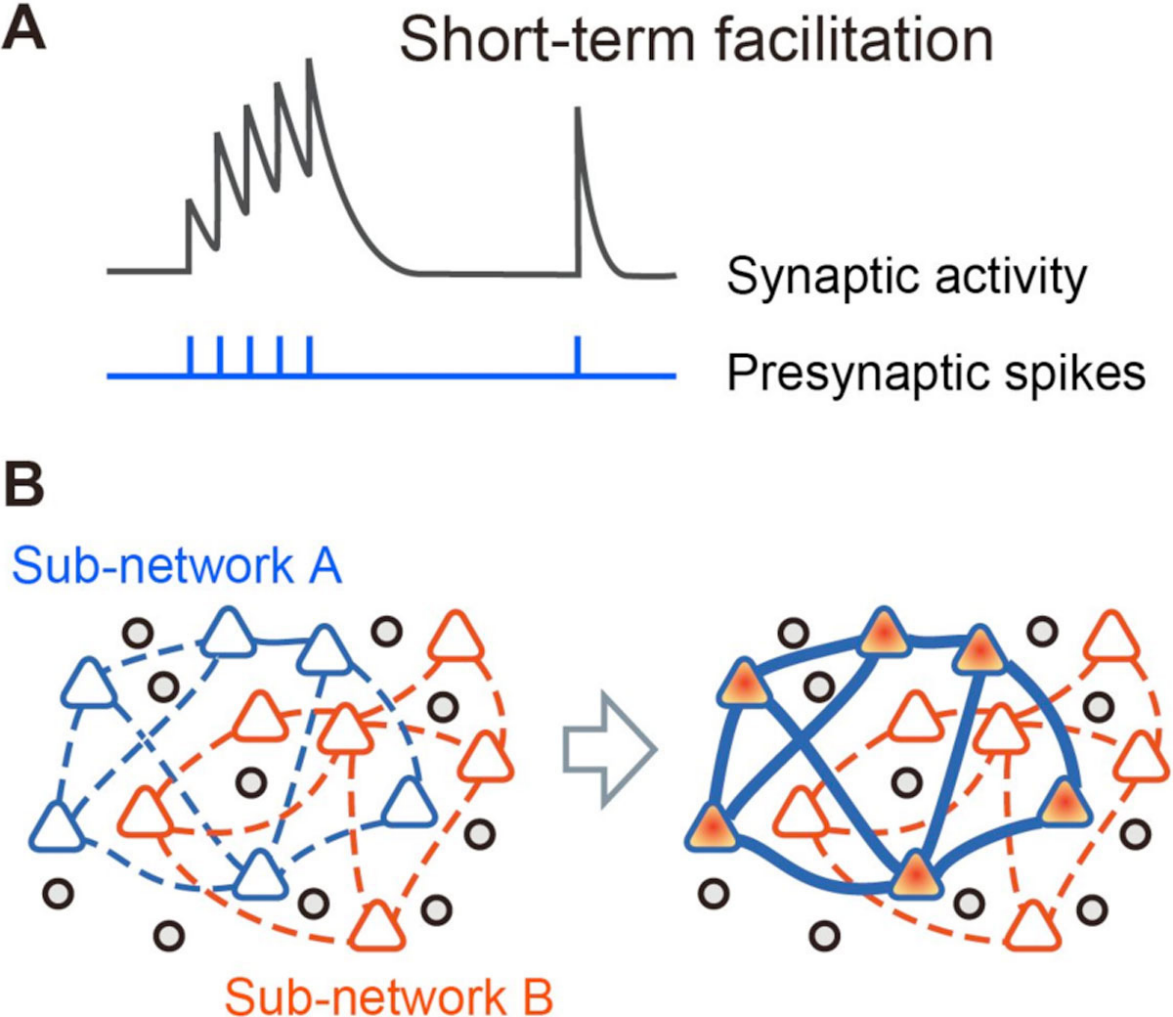
**Reorganizing network structure with dynamic synapses**. **A. **Schematic time course of synaptic activity with short-term facilitation. **B. **Depending on the activity of the network, the effective network structure changes transiently.

## Conclusion

In the neural network with dynamic synapses, the effective network structure can be reorganized; this causes qualitative changes in the population dynamics (bifurcation). In the presentation, we discuss possible network functions on the basis of this mechanism e.g., generation of sequential actions.
